# Exploring the Influence of Digitalization on Multidisciplinary Poststroke Rehabilitation Practice: Qualitative Study

**DOI:** 10.2196/77753

**Published:** 2026-02-17

**Authors:** Ann Marie Hestetun-Mandrup, Charlotta Hamre, Anne Lund, Anne Catrine Trægde Martinsen, Hong-Gu He, Minna Pikkarainen

**Affiliations:** 1 Department of Rehabilitation Science and Health Technology, Faculty of Health, OsloMet – Oslo Metropolitan University, Pb. 4 St. Olavs Plass, Oslo, 0130, Norway, 47 40674994; 2 Department for Research and Education, Sunnaas Rehabilitation Hospital, Oslo, Norway; 3 Alice Lee Centre for Nursing Studies, Yong Loo Lin School of Medicine, National University of Singapore, Singapore, Singapore; 4 National University Health System, Singapore, Singapore

**Keywords:** digital therapeutics, digital technology, goals, practice theory, rehabilitation, stroke

## Abstract

**Background:**

Leveraging digital technologies in health care is recognized as essential for effective and efficient services. However, significant challenges remain in implementing these technologies in stroke rehabilitation practice, and research on their influence is limited.

**Objective:**

This study aimed to explore the current influence of digital technologies on stroke rehabilitation practices and consider how these technologies could shape the future landscape of rehabilitation for multidisciplinary health care professionals in poststroke rehabilitation.

**Methods:**

A qualitative, exploratory design was used. Data were collected from 12 experienced multidisciplinary health care professionals at 2 Norwegian rehabilitation settings via semistructured interviews, and the data were analyzed using reflexive thematic analysis. Data analysis was guided by social practice theory.

**Results:**

The 12 participants included experienced physiotherapists, occupational therapists, speech therapists, nurses, physicians, and social workers. The following three main themes were generated: (1) Outsourcing information about and to stroke survivors: coordination and continuity within and across services (subthemes on follow-up and interservice collaboration, and user-centered approaches); (2) Navigating the ambivalence of remaining human relations in digital psychosocial support conversations (highlighting multidisciplinary challenges in building relational depth and addressing sensitive topics); and (3) Enhancing digital supplements for assessment and engagement in motor rehabilitation (subthemes on progress monitoring and motor skills exercises). Overall, the use of digital technologies in specialized stroke rehabilitation practices was seen as an adjunct to practices. While digital technologies influenced rehabilitation practices, ambivalence and challenges were noted, particularly in digitalizing multidisciplinary psychological support and exercise programs. Systems for sharing medical records and goal-setting apps, which enhance coordination and involve stroke survivors, were emphasized as future digital technologies that can shape stroke rehabilitation.

**Conclusions:**

Health care professionals used various technologies in their daily specialist practices, as well as for the coordination and follow-up of stroke survivors after referral to community services. This study identified several organizational processes, roles, standards, and rules that can act as barriers or drivers to implementing digital technologies in practice. Viewing familiar digital technology as a supplement to existing practices, rather than as a singular solution for all areas of specialized stroke rehabilitation, offers significant potential for quality improvement. These findings provide valuable insights for technology developers, health care personnel, and user groups in specialized neurological rehabilitation settings.

## Introduction

The World Health Organization (WHO) has promoted digital health to enhance the quality and accessibility of health care services [1]. Leveraging digital technologies in health care is recognized as essential for effective and efficient services [2]. Limited resources have led to earlier hospital discharge and outsourced rehabilitation services aided by digital technologies, which are areas where politicians, clinicians, stroke survivors, and researchers have turned their attention to [3]. WHO’s initiative of Digital and Assistive Technologies for Aging (DATA) [4] further underscores the importance of using accessible digital technologies in developing and sustaining new stroke care pathways [5]. The term “digital technologies” refers to electronically driven devices or applications [6,7]. Digital technologies encompass various information and communication technologies (ICTs) used in medicine and health care for disease management and wellness enhancement [6]. These technologies include devices, such as computers, smartphones, and tablets, along with their associated applications and internet connectivity [7]. Studies have shown that digital technologies improve activity and function [8-11], social participation [12], and medical adherence among individuals with stroke [13-15]. Technologies, such as gaming, videoconferencing, online health websites, apps, and wearables, have been especially explored for their potential to enhance exercise programs in both clinic and home settings in the stroke population [16]. Despite the rapid adaptation of digital technologies after the COVID-19 pandemic, scaling up remains slow [17].

Effective neurorehabilitation and tailored digital interventions are crucial for future stroke home rehabilitation [18], as they are cost-effective, portable, and motivational solutions to support daily activities [19]. However, challenges persist in home implementation [19], which might explain the lack of superior effects on supervised digital motor rehabilitation at home over conventional training for stroke survivors [18]. Although studies have found overall positive attitudes toward digital technologies in poststroke rehabilitation from the perspective of individuals with stroke, due to increased accessibility and convenience, there is an advocated need for personal contact, shared responsibility, and collaboration with health care professionals (HCPs) [20].

Building on these findings, rehabilitation specialists have introduced many digital solutions for home-based rehabilitation [21], yet significant challenges remain in adapting these technologies in practice, and research on their influence is limited. Despite guidelines and research recommending technology use in stroke rehabilitation, actual implementation and clinical validation are inconsistent [16,22-24]. A systematic review by Borg et al [25] focused on older people and people with disabilities and highlighted the need for research on technology adoption in practice, particularly from a theoretical, multidisciplinary approach. A longitudinal study across inpatient, outpatient, and community rehabilitation settings found that the type and amount of technology use varied between neurological populations, with the most frequent and varying use in stroke rehabilitation [22]. Contrary to this, Langan et al [16] found that physical and occupational therapists do not widely incorporate digital technologies into their practices, often prescribing written, paper-based, and nontechnological home exercises after hospital discharge. The primary barriers often include financial constraints, lack of technological experience, and limited time [26,27]. Despite inconsistencies in technological uptake, researchers assert that organizational readiness for eHealth reveals a dichotomy, with rehabilitation staff feeling ready, but practice preparedness being slower [2].

A broader and multidisciplinary perspective is essential to fully capture the complexity of rehabilitation practices [2,27,28]. This study addresses these opportunities for further inquiry. Existing research argues that the challenge in technology application often lies in social behavior around technology use, rather than the technology itself [29,30]. A second critical challenge lies in understanding organizational readiness for the adaptation of digital technologies in rehabilitation settings [2]. This includes the preparedness of both the organization and its members to adapt and integrate these technologies in their daily practices [2]. A third challenge is that existing studies often overlook the broader social context in which these technologies are used in practice [31]. Therefore, there is a need to consider the interconnectedness of health and social care when evaluating how digital technologies shape practices, where digital solutions should aim to lower barriers and facilitate drivers for access to rehabilitation [32].

We applied social practice theory as a theoretical framework to understand how digitalization affects stroke rehabilitation practices at the individual and social levels. Social practice theory, a behavioral theory, explores how people engage with the social world and how it impacts human behavior [33,34]. This applied practice theory is based on work by Reckwitz and incorporates elements from Bourdieu, Giddens, Foucault, and Schatzki [35]. Instead of focusing only on concepts, such as intersubjectivity, embodiment, language, and power within organizations, it incorporates all these concepts and takes a broader perspective by examining what people do in practice and the interconnected elements of individual actions and institutional structures [33]. Practice entails routine behavior involving linked key concepts, such as agents’ skills and meanings needed for practices, the things that shape and enable practices, the rules and social norms guiding practices, and the process structure [35]. This theory, which has gained recognition in various fields, provides a useful lens to address the challenges and solutions related to digital inclusivity [36]. It has been used in environmental research [37], education [33], digital information [38], and public and clinical health [31,34,39,40]. The application of this theory in poststroke settings is novel, and this study adds to the limited qualitative research on the changes in practice through digitalization [40]. This study applies the concept of digitalized stroke rehabilitation as a social practice to explore how digitalization influences rehabilitation within a local context. Therefore, this study aims to examine the current influence of digital technologies on stroke rehabilitation practices and consider how these technologies could shape the future landscape of rehabilitation. This is performed through the following research question: How do digital technologies shape the current and perceived future everyday practices of multidisciplinary HCPs in poststroke rehabilitation?

## Methods

### Design

In this descriptive qualitative study, a theoretical framework of social practice theory (henceforth “practice theory”) [35] was applied as a lens to explore and interpret the structures shaping the use of digital technologies in 2 Norwegian stroke rehabilitation settings. Inductively generated thematic themes were mapped onto practice theory concepts. This design allowed for an in-depth exploration and assessment of the relationship between various theoretical key components of the challenges and opportunities of digitalization in stroke rehabilitation, providing insights that could shape the future landscape of rehabilitation.

### Settings and Participants

Multidisciplinary HCPs from 2 rehabilitation wards in Norway were invited to participate via email and staff meetings. Recruitment at the first hospital (June 2022 to January 2023) and the second hospital (April to May 2024) involved multidisciplinary HCPs, facilitated by team coordinators. Purposeful sampling was used to recruit appropriate and information-rich data for the in-depth qualitative study. Participants were selected for their direct experience with multidisciplinary specialized stroke rehabilitation. There were no a priori guidelines for the amount or the specific data required, such as regular engagement with new technologies. The study started broadly and narrowed naturally as we became interested in what qualifies as specialized stroke rehabilitation and how technologies shape practices, justifying the theoretical delineation of the sample [41]. Both settings specialize in poststroke rehabilitation, regularly engaging with new technologies. Some technologies have been implemented in practice, while others are still being tested or are under consideration.

The first rehabilitation hospital is a large specialist facility with subacute and poststroke wards, allowing for both short- and long-term rehabilitation. Recruitment occurred in the poststroke ward, with an average of 10 days poststroke at admission and an annual average of 279 stroke survivors admitted. The second ward is a community-based clinic within a hospital and focuses mainly on subacute stroke rehabilitation, with an average of 32 days poststroke at admission and an annual range of 150‐200 stroke survivors admitted. Despite differences in organization, both settings were chosen for their high staffing levels for specialized and intensive multidisciplinary stroke rehabilitation. The first hospital qualifies as performing complex rehabilitation due to the involvement of at least six different professions. Maximum variation purposive sampling [42] was used to recruit 12 experienced HCPs, ensuring diversity in age, sex, and work experience (Table 1). Participants had at least 2 years of experience in poststroke rehabilitation, and both female and male professionals were included. Interviews were conducted on-site at the hospital during appropriate times to minimize interference with daily practices.

**Table 1.** Demographic information of the health care professionals who participated in this study (N=12).

ID	Profession	Age (years)	Time working in stroke rehabilitation (years)	Workplace and rehabilitation practice	Current digital technologies used in rehabilitation
1.1	Speech therapist	31	2‐5	Specialized stroke rehabilitation hospital	Videoconference, digital speech training, video fluoroscopy, and electronic medical records
1.2	Occupational therapist	35	5‐10	Specialized stroke rehabilitation hospital	Videoconference, pilot project on stroke survivor access to medical records, QR codes to health-related webpages, robotic technology training, digital group motor rehabilitation, and electronic medical records
1.3	Physiotherapist	30	2‐5	Specialized stroke rehabilitation hospital	Videoconference, virtual reality lab, and electronic medical records
1.4	Occupational therapist	38	5‐10	Specialized stroke rehabilitation hospital	Videoconference, webinars, virtual reality, and electronic medical records
1.5	Nurse/head of the ward	42	5‐10	Specialized stroke rehabilitation hospital	Videoconference, virtual reality lab, robotic technology training, and electronic medical records
1.6	Nurse	53	2‐5	Specialized stroke rehabilitation hospital	Videoconference and electronic medical records
1.7	Physiotherapist	52	25‐30	Specialized stroke rehabilitation hospital	Videoconference, web-based exercise programs, and electronic medical records
1.8	Social worker	40	5‐10	Specialized stroke rehabilitation hospital	Videoconference, national health webpages, electronic individual plan, and electronic medical records
1.9	Physician/head physician	57	15‐20	Specialized stroke rehabilitation hospital	Videoconference, webinars, and electronic medical records
2.1	Physiotherapist	54	<30	Rehabilitation clinic within a hospital	Videoconference, pulse monitoring, digital blood pressure equipment, digital exercise program, and electronic medical records
2.2	Physiotherapist	35	5‐10	Rehabilitation clinic within a hospital	Virtual reality, videoconference, pulse monitoring, step counter, iPad, dynamometer, and electronic medical records
2.3	Physiotherapist	36	5‐10	Rehabilitation clinic within a hospital	Web-based exercises, pulse monitoring, dynamometer, biometric equipment, Nintendo Wii, step counter, and electronic medical records

### Theory

In this study, practice theory offered a lens through which to examine how different HCPs talked about their actions. Grounded in constructionism, the theory posits that learning and knowledge are collectively produced through shared understandings, practices, and language [33]. Practice theory situated digitalization in poststroke rehabilitation within 2 practice settings, and its terminology served as an analytical advancement for understanding how digital technologies influence poststroke practices. Its key concepts are mapped in Table 2 to clarify the description of each concept as it relates to specialized poststroke rehabilitation settings.

**Table 2.** Glossary of practice theory concepts related to poststroke rehabilitation practice.

Concept	Practice theory concepts	Example related to poststroke rehabilitation practice
Agent (including skills)	An agent is a carrier of actions with the necessary skills, and this encompasses both bodily and mental routines. It involves ways of understanding the social world and implicit and explicit know-how [35].	Health care professional agents are connected through specialized hospital processes [43] and are norm-following individuals shaped by professional know-how, who adhere to stroke guidelines and hospital policies relative to their hierarchy at the hospital [40]. They use their minds and bodies to perform routine actions during stroke rehabilitation, such as instructing stroke survivors on walking while encouraging progress. In both contexts, they interact with medically stable stroke survivors past the acute phase, when they are cleared for intense rehabilitation. Occupational therapists, physiotherapists, nurses, medical social workers, and speech and language therapists are central to practices when motor rehabilitation is the main focus [43].
Things	To carry out a practice, agents use things in a certain way, such as physical entities or material conditions, where actions take place. However, writing, printing, and electronic media are also a part of things [35].	Things represent all therapeutic devices, equipment, and health solutions that are necessary to carry out the practice of stroke rehabilitation. Physical elements, such as conversation rooms and rooms for exercise or written material, encompass the things that the agents use or interact with [35].
Rules	Structural rules construct a practice around communication chains and signs. Agents endorse cultural norms and meanings to objects, determining actions through routinized nonsubjective understanding [35].	Health care professionals in poststroke rehabilitation follow recommended interventions from recent rehabilitation guidelines and national clinical practice guidelines for stroke rehabilitation [43]. Communication between professionals often occurs via health information systems, electronic medical records, and IT platforms where rehabilitation plans, progress notes, and patient outcomes are documented and coded according to standardized protocols. Communication with patients or their families typically happens through face-to-face consultations, telephone follow-ups, or digital platforms for remote monitoring, ensuring adherence to evidence-based practices [40].
Process structure	Routinized social practices and processes occur in a sequence over time and through repetition, forming the core of the social structure. Changes in practices arise internally, as practitioners challenge or resist established routines, and externally, as intersecting practices influence each other. Practitioners improvise new actions in novel situations, prompting internal shifts, while external interactions among diverse practices foster adaptation and transformation [35].	In poststroke rehabilitation, health care professionals facilitate a process-centered rehabilitation model for stroke survivors. Although structured, it starts flexibly with initial consultations, followed by planning, a discharge meeting, and ultimately self-care. The process includes multidisciplinary subprocesses like therapeutic examinations and collaboration with other institutions [44].

### Data Collection

Individual semistructured interviews were conducted over 2 years, from 2022 to 2024, each lasting 40 to 60 minutes. Information power guided the inclusion of 12 interviews, based on data relevance and richness [45] and when sufficient data “told a coherent, convincing, and comprehensive story” [41]. The interview guide, collaboratively developed by the study’s authors (Multimedia Appendix 1), explored participants’ stroke rehabilitation practices, shared decision-making, and the use of technology and future digital innovations. The inclusion of decision-making as a part of the stroke rehabilitation process was inspired by its person-centered foundation, drawing on literature by scholars, such as Rose et al [46] and Bomhof-Roordink et al [47], in formulating interview guide questions. Interviews were conducted in Norwegian, allowing for probing questions and guided conversations. Audio recordings were securely stored using services for sensitive data. A transcription software assisted with transcription to Norwegian, which was later manually edited for accuracy. Deidentified selected quotations were then translated into English and checked for accuracy by the research team and a secure university-based artificial intelligence (AI) tool (ChatGPT-4, OpenAI). The research team retained responsibility for final wording and meaning.

### Data Analysis

Thematic analysis was chosen to identify, analyze, and interpret patterns across varied meanings [48]. This approach is well-suited as a method for exploring complex phenomena, as it allows for a rich and nuanced understanding of the HCPs’ perspectives on how they construct and navigate their practices. The flexibility of thematic analysis enables it to be effectively applied across diverse theoretical frameworks and research questions, making it appropriate for this study’s aim of understanding multidisciplinary HCPs’ practices. We used reflexive thematic analysis (RTA), specifically developed by Braun and Clarke [48], because it aligns with the interpretative paradigm, emphasizing the active role between researchers and interviewees in generating knowledge and the subjective, situated nature of data interpretation. The in-depth engagement with the data enabled us to explore the social, cultural, and contextual complexities of digitalization in stroke rehabilitation practices, as well as the iterative and reflective process of capturing the underlying meanings and nuances of HCPs’ perspectives [48]. The 6 phases of RTA—familiarization, coding, theme generation, review, refinement, and write up—were applied in a back-and-forth manner to accommodate curiosity and interpretation. Familiarization and coding were performed in NVivo (Lumivero), while theme generation, refinement, and write up were conducted in Word (Microsoft Corp). We conducted 2 analyses. The first analysis followed RTA, and the second aligned the findings according to practice theory concepts using Excel (Microsoft Corp). Both descriptive (“semantic,” according to Braun and Clarke [48]) and interpretative (“latent”) codes were utilized [49] with emphasis on capturing the empirical data during the coding stage. After several rounds of coding, the codes were manually organized into main themes, with practice theory guiding further analysis. The themes from the RTA did not change in meaning, but further descriptions and interpretations related to themes and subthemes were inspired by practice theory as a lens. During theme refinement, an additional analysis mapping qualitative data according to practice theory’s 4 concepts was conducted [35] (Multimedia Appendix 2). Practice tasks, forming the final subthemes, were defined. An example of the six phases of RTA, including analyses from both RTA and practice theory, is presented in Multimedia Appendix 3. Key concepts are written in italics in the Results section. Each concept was examined from the points of both the current and future states to understand how digital technologies shape HCPs’ practices.

### Reflexivity

The researchers’ backgrounds acknowledge how the authors’ positionality influenced data analysis [48], with several having clinical experience with stroke survivors (AMH-M, CH, AL, and HGH). All authors have academic backgrounds, experience in digital solutions, and multidisciplinary expertise in the fields of rehabilitation within occupational therapy, physiotherapy, nursing, computer science, and medical physics and technology. The first author, a doctoral student and experienced physiotherapy specialist, conducted all interviews, considering subjectivity to be a resource in conducting and analyzing data; maintained a listener’s role; and emphasized her role as a researcher rather than an HCP. Still, her clinical background allowed her own experiences to resonate with participants, enabling her to listen and repeat participants’ recognizable stories. Reflexivity was considered early in the analysis process by recording initial assumptions after each interview and writing memos and annotations in NVivo during coding to ensure the quality of analytical points and the possibility of reviewing interpretations. A description related to all codes also supported analytical interpretation. To ensure rigor, the authors collaborated in regular meetings and workshops to discuss and enhance understanding, develop comprehensive descriptions, and refine the analysis, strengthening confirmability, coherence, and clear theme boundaries [48,50].

### Ethical Considerations

As the study’s focus on health service research fell outside the scope of the Health Research Act § 2, ethical approval from The Norwegian National Research Ethics Committee for Medical and Health Research was not required (reference number: 279418). Approval was granted by the Norwegian Agency for Shared Services in Education and Research (reference number: 668899) to ensure privacy protection and adherence to ethical standards. Participants received oral and written information about the study, and written informed consent was obtained prior to the interviews. The first author reiterated consent form details, emphasizing the voluntary nature of participation, confidentiality, and the participants’ right to withdraw from the study. Participants did not receive any compensation for their involvement in the study.

## Results

### Themes and Subthemes

The results are presented in themes and subthemes and exemplified with selected quotes extracted from the analysis based on practice theory.

### Theme 1: Outsourcing Information About and to Stroke Survivors: Coordination and Continuity Within and Across Services

#### Overview

HCPs actively leveraged digital technologies to enable better coordination and continuity of care across services. Digital tools facilitated collaboration not only between HCPs in cross-service collaboration but also with stroke survivors, relatives, and interpreters, forming the first subtheme. Through their practices, they sought to harness digital health to streamline communication and improve rehabilitation outcomes, despite the challenges of sustaining structured coordination. Maintaining continuity after referrals to community services was challenging, requiring constant goal adjustments and balancing person-centered approaches. The rule of involving stroke survivors in their rehabilitation was viewed as a necessary but complex procedure, reflected in the second subtheme “navigating user-centered approaches to rehabilitation for stroke survivors in the digital era.”

#### Subtheme 1.1: Follow-Up and Continuity in Rehabilitation in Collaboration With Stroke Survivors and Between HCPs in Cross-Service Collaboration

In their current practices, HCPs use digital technologies, primarily videoconferencing, for stroke follow-up after discharge, involving additional *agents* such as stroke survivors, community HCPs, interpreters, Norwegian Labor and Welfare Administration (NAV) advisors, and relatives. The importance of HCPs with ICT knowledge as an added skill was highlighted. Coordination and continuity were perceived as crucial owing to the challenges in maintaining structure after referrals to community services, but they often relied on the agency of stroke survivors.

*Keeping track of who to contact and when is challenging. We’ve faced logistical issues regarding when the reablement team is supposed to come, and when it conflicts with orthopedic appointments. We lack an overview of these things when the stroke survivors aren’t hospitalized and rely on information from stroke survivors or other responsible persons. We do have good contact with some teams, but the organization varies greatly between different districts, making it very inconsistent.*

[Informant 2.2]

Increased coordination and continuity aligned with the explicit *rules* of increasing outpatient activity and outsourcing information about stroke survivors. For future practices, an HCP suggested creating a unified individual digital plan between specialist services and communities to address uncertainty about digital progress. This system would provide an overview and ensure that individuals needing long-term, coordinated services receive appropriate care and benefit from effective collaboration to meet their goals. The following comment was made:

*It would have been quite easy to get started with creating the document and then pass it on to the community. I know that there is no common solution, because it is somewhat up to the community whether they have a digital individual plan, and at least it was a few years back, so I don't know if there is a common solution that all communities can use, but if there is, then there is great potential to look into it.*

[Informant 1.8]

Others proposed sharing medical records reporting standardized test results across services and using apps with push-warnings for goal-setting visible to both the HCP and the stroke survivor. Future solutions could involve extended digital therapy skills to include other digital platforms, such as NAV’s screening tools, forming a new type of *agent* bridging HCPs and NAV advisors. They also proposed a system between the stroke survivor and health care provider to navigate in-hospital appointments and communicate with staff.

*Maybe an overview for the patient, in an app, or some kind of guide, showing you this way, you can follow this path to get there. Your physiotherapist is sick today, so your appointment is cancelled. This is somewhat similar to what has already started in other wards. We will also implement it here eventually. It just needs to be adapted to our ward.*

[Informant 1.6]

There were societal encouragements for increasing the outpatient activity and outsourcing information to the stroke survivor, enabled by more videoconferencing. Closer follow-up and a more standardized program under the direction of either a general practitioner (GP) or hospitals were mentioned. Enhanced outpatient activity demanded restructuring practices, creating better access to stroke-related information, and introducing a joint communication system between the stroke survivor and the HCP. Digitalizing content on stroke exercise programs, fatigue, rehabilitation experience forums, and cognitive or physical constraints was suggested for improved coordination. However, the complexity arising from multiple medical record systems was highlighted, showing that these systems were used exclusively between HCPs and GPs, leaving out stroke survivors. This suggests the need for integration through a unified platform for visibility and collaboration.

Even though collaboration between HCPs across health services is essential, they expressed a lack of knowledge about available resources for stroke survivors outside their own settings, underscoring the role of stroke survivors in informing their own GPs about rehabilitation opportunities. Paper-based reports were still the main communication tool. Clarifying realistic expectations and outcomes for the stroke survivor’s condition helped in establishing routines and maintaining standards and rehabilitation plans. To enable this, HCPs routinely used SMART (specific, measurable, achievable, relevant, and time-bound) goals and educated stroke survivors in SMART goals [51] based on the understanding of being *specific or measurable*, *achievable or attractive*, *realistic*, *and time-bound* (informant 1.5), which gave direction for the future rehabilitation process. However, HCPs expressed that stroke survivors’ goals, as revealed in goal-setting conversations and documented in written reports, were often forgotten or changed. The flow of information heavily depended on the stroke survivor’s effort to communicate with other HCPs. As a future extension of implementing SMART goal principles in home-based rehabilitation, some HCPs perceived that goals could shift to being more focused on daily routines rather than solely the therapeutic understanding of goals for stroke survivors. For future development, HCPs valued apps with push notifications for goal-setting visible to both HCPs and stroke survivors, and indicated that future practice could consist of increased health-related data and goals. The following comment was made:

*I wish we could have a bit more follow-up along the way, mostly for the stroke survivor’s sake, because it’s quite easy to put that binder in a drawer when you get home. Then you have, in a sense, "free time" until they come back. Since we say that goal setting is so important, I wish we could have communication along the way....If they could almost get a push notification through an app*.

[Informant 1.2]

Collaboration faced time constraints in cross-service communication, and HCPs highlighted the importance of “setting boundaries for oneself” (informant 1.4) to balance their workload and reduce personal-professional responsibilities. This perspective was contrasted by another participant, who highlighted differences between specialist health care services and communities in care provision and time allocation, noting the challenges in aligning social expectations.

*I imagine that in the communities, you might get a couple of assessment visits and then wait a few months for some assistive devices, and you might not get them. You certainly don't get anything weekly and can only address one thing at a time. If we propose ten measures, it might be that you can either spend ten minutes per measure spread over a few weeks, or half of the measures are left out, is the impression I have*.

[Informant 1.2]

Conversely, an HCP made the following comment: “We can’t really demand that the community follow up on everything we do either” (informant 1.5). Some HCPs desired more follow-up time in their practices due to a known hesitancy in working with stroke survivors in communities, suggesting more knowledge on stroke rehabilitation and restructuring practices for better access to information and logistics. The potential benefits of a joint communication system include a less fragmented goal process and user involvement in rehabilitation goals for stroke survivors and their relatives.

#### Subtheme 1.2: Navigating User-Centered Approaches to Rehabilitation for Stroke Survivors in the Digital Era

Stroke survivors were routinely involved in their rehabilitation through discussions at several rehabilitation meetings, aiming to foster independence and proactive behavior. Digital preassessment conversations encouraged participation of stroke survivors in decision-making. The *rule* of involving stroke survivors in rehabilitation was to address their existential needs and was echoed by HCPs as fundamental to the nature of rehabilitation. They emphasized constant goal adjustments and the balance of person-centered care as a common but complex procedure. For instance, cognitive (eg, cognitive fatigue) or cultural determinants could undermine their accountability.

*But of course, there are quite a few cognitive difficulties that cause significant disruption, which also means that if there are psychiatric and other conditions, stroke survivors may resist some of the services offered or do not take the initiative. It is often said that they are not motivated, as many lose due to cognitive impairment or other conditions, the ability to motivate themselves or make decisions and take initiative. And they often fall behind.*

[Informant 1.9]

Relatives were involved, and they were invited to meetings either in person or remotely. HCPs supported stroke survivors’ relatives by providing written material for support, owing to their role of being both a close relative and a caregiver. The challenge of maintaining this balance was highlighted as follows:

*It’s a challenge for relatives to live with someone who has had a severe stroke and to be both the closest relative, personal trainer, and everything else. They may need to be a home helper, assist with meals, and serve. It’s a significant burden. It is beneficial for them to have someone come in who can take on some of the burden.*

[Informant 2.1]

HCPs initiated local opportunities to continue rehabilitation, often referring stroke survivors to other services, acknowledging the complexity of stroke rehabilitation where “most need more rehabilitation” (informant 1.7). Many stroke survivors returned to the same rehabilitation hospital owing to gaps in service availability, such as occupational therapy lacking in some communities. Future digital rehabilitation would require stroke survivors to be more involved in discussions about rehabilitation opportunities throughout the process and to take ownership, which are often omitted in current practices. The following comment was made:

[In digital communication with stroke survivors it’s important to] *provide alternatives because there’s not just one option; there are often several factors to consider. So, it’s also about involving the stroke survivor more actively in the discussions about their future as well.*

[Informant 1.6]

Concerns were expressed about current routines that excluded stroke survivors from discussions regarding alternatives in rehabilitation. HCPs viewed passivity after stroke and a lack of insight and comprehension as barriers to increased agency. However, HCPs observed a growing trend of stroke survivors taking an active role in their treatment. Although this was problematic for most owing to a lack of comprehensive medical and rehabilitation knowledge, there was a clear need for enhanced stroke survivor education. The potential for increased use of digitalized materials for relatives was also suggested. However, some HCPs criticized the assumption that most people are digitally proficient, noting that this responsibility often falls on not only stroke survivors but also their relatives. In situations where stroke survivors struggle to learn new skills, the assistance of relatives was seen as essential. The following comment was made:

*Not all stroke survivors can use digital solutions, and then [the responsibility] might fall to their relatives. However, not all stroke survivors have resourceful relatives either, creating ambivalence about whether this user group can manage it. I believe that a large proportion can, whether it’s the stroke survivors or their relatives who take responsibility. As shown in videoconference meetings here, where relatives have participated, most manage if they get a brief introduction to what they need to do, how to do it, and when. Many will be able to be part of such an arrangement in the long term, but it is probably a long way to get there.*

[Informant 1.3]

Education was needed for stroke survivors with cognitive difficulties or limited digital skills. High demands were placed on those facilitating digital interactions. There were divided opinions on whether the rehabilitation hospital or the community held the main responsibility for digital home exercises and follow-ups. Some claimed that districts were responsible for digital home rehabilitation and follow-up, while others believed that the hospital’s main future task was to spread and utilize their specialist expertise. Nonetheless, the user’s capabilities to become digitally self-sufficient were questioned, and an individualized approach was the top priority. The following comment was made:

*So, when we say that we aim to provide equitable services to our users and stroke survivors, we must also remember that equitable can mean that some need in-person appointments while others can manage with digital solutions. There are many ahead of us in digital communication, including users and partners, and we are somewhat behind. But many are also far behind us and will never reach where we want to be, so we must remember to cater to the full range of our users. We cannot demand that everyone go digital. If we do, it must be very much based on collaboration. Maybe they can manage an app, but not video. We need to find the right levels.*

[Informant 1.2]

### Theme 2: Navigating the Ambivalence of Remaining Human Relations in Digital Psychosocial Support Conversations

Multidisciplinary HCPs played a crucial role in providing psychological support through communication, but grappled with the dual promise and limitation of digital technologies in providing support. Psychosocial support remains a multidisciplinary task as a person-centered rehabilitation approach often frames these practices. They engaged in both structured and unstructured conversations to assess existential struggles, self-perception changes following stroke, and quality of life, especially for stroke survivors with multiple cognitive and psychiatric challenges. These conversations often focused on enhancing well-being, and the rehabilitation *process* was about gaining knowledge about the difficulties and the approaches to tackle them.

While digital communication enabled flexibility and accessibility, providing effective support still required building trust and relational depth. HCPs emphasized therapeutic communication skills involving active listening, openness, and sharing, capturing sensitive information in both digital and in-person meetings. Conversations sometimes included topics like depression or suicidality, but often, these were viewed as expressions of an unwanted situation rather than an active plan. The significance of building a relationship with stroke survivors digitally was highlighted through practical strategies to achieve it.

*Just giving them the time and space to express themselves, joking a lot. I use a lot of humor with the stroke survivors and try to show some understanding of their situation. But I find that if you can mention relevant life experiences, I think it’s a good way to build relationships, to dare to open up a bit, so we're not just sitting there as rigid authority figures, making it a one-sided conversation.*

[Informant 1.1]

Some HCPs felt that building relations is harder to achieve digitally and raised concerns about digital health potentially diminishing their therapeutic skills, emphasizing the importance of human relationships over relying solely on technology.

*I think one should be a bit cautious about saying that all therapists are now redundant. Because now they can just train with an app. I don't believe in that. It’s about human relationships.*

[Informant 2.1]

This ambivalence was further shaped by the need to balance technological efficiency with the irreplaceable value of human connection. As professionals sought to navigate the tension between digital and in-person care, building relationships digitally was seen as a prerequisite for further effective support, particularly for follow-up in the later rehabilitation stages.

*These conversations, right, which many experience as being more difficult- yes, life can become challenging when you return home. The challenges where perhaps there could also have been more follow-up conversations via video, but also something in a group setting. We currently have groups for those who come here, but the number of participants is somewhat limited Many live far away, and it’s difficult for them to travel from home. So, perhaps there should be a type of service specifically directed at stroke survivors and their relatives at a later stage.*

[Informant 1.9]

### Theme 3: Enhancing Digital Supplements for Assessment and Engagement in Motor Rehabilitation

#### Overview

HCPs embraced digital tools to enhance assessment, treatment, and patient engagement in motor rehabilitation. These tools provided detailed insights into physical progress, fostering participation and curiosity about their health metrics, as shown in the first subtheme “assessment and monitoring of progress.” HCPs emphasized individualized approaches and explored creative solutions, such as using virtual reality (VR) and augmented reality (AR), to simulate real-life scenarios or design exercises based on videos in home environments, as illustrated in the second subtheme “exercises and rehabilitation programs targeting motor skills.” However, digital tools also presented challenges, such as balancing safety concerns for patients with poor balance and ensuring engagement across generations. While digital tools offered significant potential for self-managed and supported rehabilitation, they were best suited for less complex cases.

#### Subtheme 3.1: Assessment and Monitoring of Progress

HCPs used various digital tools to enhance the assessment of stroke survivors’ progress and engagement in therapeutic activities. *Things* included pulse monitors, dynamometers with apps to measure and track physical strength and progress, video-based assessments like video fluoroscopy and endoscopic swallowing evaluations, VR tools like “Job Simulator” for cognitive assessments involving kitchen tasks, and step counters that display progress graphs. This fostered curiosity and provided detailed insights into treatment intensity. For instance, pulse monitors, which have been valued as indispensable over the past years, ensured that the pulse rate of stroke survivors remained within a target range.

*We measure the pulse and check that we are in the zone as much as possible....And after using it for a few years now, I kind of don't understand how I managed without it. It says a lot about the patient’s intensity.*

[Informant 2.1]

Multidisciplinary assessment was integral to the *processes* of specialized stroke rehabilitation*,* focusing on holistic understanding and ongoing evaluation, which directly influenced HCPs’ replicated behavior and their ability to act on it.

HCPs proposed using digital tools for preadmission consultations to evaluate engagement and proposed involving more community therapists, with local resources aiding preassessment. They suggested video-based visualizations of stroke survivors’ home environments and the storage and processing of stroke survivor assessments. Monitoring was perceived as identifying stroke survivors in need of more interventions, but motivation and performance remained challenges. The following comment was made:

*Monitoring by itself will not be enough, because when you look at the studies that have been done, at least in my experience, stroke survivors, even when they improve in arm function and actually have a higher capacity, don’t use their arm as much when they get home. They revert to their usual patterns. Even though capacity increases, performance does not necessarily increase. This is a challenge that the monitor can detect, but the monitor itself will not address the stroke survivor’s motivation, incentives, or desire to use the arm, or their understanding that they probably can walk more or use the arm more than they currently do.*

[Informant 2.3]

Integrated feedback systems within the monitoring process were seen as potential solutions to motivation challenges. Nonetheless, the question of whether the encouragement of therapists alone is sufficient to enhance arm use or gait remains unresolved, and HCPs are yet to find effective solutions.

#### Subtheme 3.2: Exercises and Rehabilitation Programs Targeting Motor Skills

Both hospitals implemented gamified rehabilitation. In one hospital, VR was used for intensive balance training or as part of constraint-induced movement therapy for arm training, in addition to being utilized during digital group sessions. The other hospital used biometric equipment with force plates or the Nintendo Wii device in practice and VR for projects. One HCP had experience with asynchronous virtual individual exercises for stroke survivors living at home, when working in the community.

HCPs provided assistance either in person or remotely during the digital programs, but VR sessions required their presence. Language training, in which stroke survivors underwent naming therapy and practiced word recall, was more effective with an HCP present. *Rules* on therapy dose varied, but quantity over quality was mainly emphasized, with the intention of promoting cerebral blood flow and brain reorganization.

Despite efforts to fully utilize each stroke survivor’s rehabilitation potential, challenges in maximizing their potential persisted. The following comment was made:

*You really want that person to have the interventions implemented so they can achieve good functionality. It’s very, very sad if there’s potential that isn’t fulfilled. Then our efforts here feel somewhat wasted. We can figure out and write down what is needed, but if it’s not followed up, it feels a bit pointless. I just want people to get as well as they possibly can.*

[Informant 1.7]

To enable greater potential for rehabilitation, HCPs proposed future practices involving goal-oriented digital rehabilitation opportunities and motivating exercises. However, they highlighted variations in the types of stroke survivors suitable for undergoing digital motor rehabilitation. As a result, the tasks of future HCPs would include designing motivating exercises while ensuring environmental safety and space. HCPs noted that poor balance and fall risk complicated independent exercise, making approaches to individualized adaptation and treatment effectiveness critical. Simple, self-managed technologies were seen as valuable for catering to different generations. For example, sitting exercises for arm rehabilitation were favored among HCPs owing to safety concerns, especially given that arm rehabilitation tends to receive less focus in stroke rehabilitation across services. Rehabilitation programs could be self-managed or supported by HCPs, depending on the complexity of the stroke survivor’s condition. Moreover, they suggested the use of digital apps for low-threshold language training, incorporating features like animations, facial expressions, and tongue position guides. Other HCPs saw potential in creating videos of stroke survivors’ homes to design VR or AR rehabilitation programs that simulate real-life challenges and create virtual training apartments, enhancing the transferability of rehabilitation practices to the home setting. Despite some doubts, many saw the potential of digital technologies to improve rehabilitation practices. The following comment was made:

*Stroke survivors tend to walk very little. Many of them move very little. When they return home, they might train or have follow-up sessions with a physiotherapist maybe two, three days a week, at most. Some might attend a day center, but their overall movement remains limited. In that sense, if there were a low-threshold alternative to encourage them to get up and engage in physical activity would be beneficial.*

[Informant 2.2]

## Discussion

### Principal Findings

**Figure 1.**
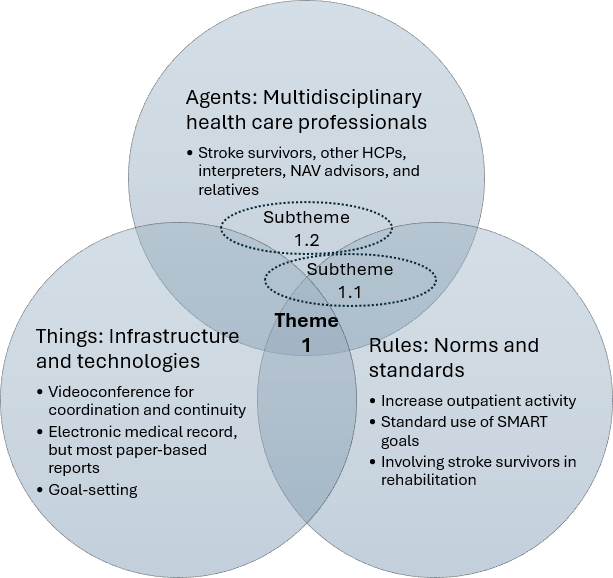
Diagram illustrating an example of the first overarching theme (with subthemes) and its relationship with social practice theory concepts. HCP: health care professional; NAV: Norwegian Labor and Welfare Administration; SMART: specific, measurable, achievable, relevant, and time-bound.

This study explored how digital technologies shape the current and future practices of multidisciplinary HCPs in poststroke rehabilitation. According to the results and as linked to practice theory concepts illustrated in Figure 1, the primary agents (HCPs) collaborated with various other agents, including stroke survivors, relatives, interpreters, community HCPs, and NAV advisors, during their practices.

HCPs were influenced by a range of digital technologies, particularly videoconferencing, for rehabilitation coordination and continuity, although paper-based reports remained the primary communication tool across services and with stroke survivors. They used digital instruments for assessment and engagement, including gamified exercises. However, there was ambivalence in using digital technologies for multidisciplinary psychosocial support owing to concerns about their impact on human relationships.

Researchers have highlighted that contextual factors shape technology uptake, including professional discipline, rehabilitation settings, and device features [52]. The use of social practice theory helped us realize and identify how digitalization intersects with particular contextual factors that have not been emphasized in earlier stroke research, which can influence the digitalization of specialized poststroke rehabilitation practices. For example, this theory illuminated the relational dynamics between HCPs and stroke survivors by directing our attention to the required skills and know-how, as outlined in Table 2. Specifically, with regard to theme 2, this perspective highlighted how trust and relational depth are integral to the integration of digital tools. Additionally, this lens revealed how institutional structures, such as differing medical record systems and the digitalization of support conversations, altered established practice routines and were sometimes challenged or resisted by HCPs. Drawing from these core concepts and the results, this study highlights that the primary agents need digital therapy skills, potentially expanding to include knowledge of other digital platforms in cross-service collaboration. Previous research has shown that digital tools, such as apps, can give HCPs a better overview of stroke survivors’ rehabilitation processes and can help in follow-up after discharge [53], particularly by using existing digital technologies in transitions between services. However, transitions between specialist health care and community-based services were found to be challenging in this study, requiring familiarization with different practices. Extended use of digital technologies could bridge this gap by enabling therapists to familiarize themselves with different practices during the coordination and continuity of rehabilitation. HCPs highlighted future unified digital systems for sharing medical records and test results across health care services, with goal-setting apps visible to both HCPs and stroke survivors. These could enhance coordination, user involvement in rehabilitation goals, and more active stroke survivor participation and agency in digital follow-up. Despite claims of low technological uptake among therapists [16], other longitudinal studies have found that in a multidisciplinary team, occupational therapists and physiotherapists used digital technologies more frequently across inpatient, outpatient, and community rehabilitation settings. However, studies agree that advancements are needed to match the rehabilitation context, including specific technology training and guidance of implementation [22]. Related responsibilities regarding whether the rehabilitation hospital or the community should lead this practice reorganization were found to be confusing among HCPs in this study. Additionally, they proposed future digital rehabilitation innovations visualizing stroke survivors’ home environments and involving more community therapists while addressing safety concerns in stroke rehabilitation exercises. The rehabilitation process usually comprises the following 4 steps: assessment, goal-setting, intervention, and evaluation [54-56]. The results of this study relate to all aspects of rehabilitation and illustrate the complexity of how digitalization is shaping specialized stroke rehabilitation practices.

Consistent with our findings, other qualitative research has indicated that using digital technologies adds value in coordination and continuity, especially where user involvement is lacking. Comprehensive care coordination programs, including home telehealth, can aid stroke survivors and their relatives in managing stroke recovery through weekly real-time video calls and in-home messaging [57], enhancing accountability and self-management [58]. Moreover, tablet-based therapeutic programs can provide alternative access to rehabilitation services and follow-up. Studies have found positive attitudes among other HCPs toward digital apps for sharing stroke rehabilitation information with stroke survivors and their families [53]. However, researchers have highlighted the importance of infrastructure, equipment, space, and support in adapting telerehabilitation [17]. While ICT platforms facilitate communication and effective goal-setting among stakeholders in the rehabilitation process [32], they should also provide feedback to support the interest and progress of individuals with stroke [58,59], which is often limited or not adequately provided in current practices, as observed in this study. However, a range of digital technologies have been found to be integral to self-management for stroke survivors in the rehabilitation process, suggesting that these technologies can prevent therapists from taking over before users are able to do things themselves [20]. Nevertheless, both our findings and the findings of existing research have revealed a distinction between the therapeutic understanding of goal effectiveness and actual digital rehabilitation activities. Resistance to trivial digital technologies offering only engagement has been noted, and there are opportunities to target stroke survivor–specific goals and functional outcomes [53].

The exploration of how digital practices manifest within written documents and in community practices could be valuable, as discharge summaries are important for communication between health care levels and for ensuring continuity of rehabilitation [60]. There is potential for digitalizing written reports, and future research should be performed in this area. Although written reports are widely used, which is consistent with the findings of other studies, they lack the ability to facilitate both adherence and feedback [53]. While this was highlighted by the HCPs, it remains unclear whether therapists’ decision-making regarding new technological infrastructure is constrained by institutional requirements of productivity, and if so, to what extent [61]. In this study, paper-based reports contrasted with the incentives of outsourcing digital information. Studies have found that stroke discharge summaries often report on activities of daily living and sensorimotor and general cognitive functions, but they frequently omit stroke survivors’ needs and goals [60], and this is likely to increase with further digitalization and user feedback.

Our results contribute to the debate on whether technologies conflict with care and relationship building, emphasizing the need to transfer traditional communication skills, such as empathy, to digital platforms [62]. Claims about jeopardizing interpersonal connections due to being lost in data or a lack of in-person interactions have been made [63-66]. One reason technology may conflict with care is the limited sensory input it provides, highlighting the distinction between the critical social dimensions of humans as social sensors and some technologies as environmental sensors [67]. Despite this limitation, it has been found that using technologies can balance the roles toward a more symmetric relationship compared with the classic asymmetric stroke survivor-caregiver and HCP relationship often found in hospitals [62]. Digital platforms for collaboration, shared decision-making, and data storage can add value to a person-centered rehabilitation process [32], which, despite technology uptake, still drives rehabilitation and remains the rule of practice [52]. Video-based technologies enhance the ability to strengthen confidential and honest communication [62], but they present ethical [24] and privacy considerations for family members, involve sharing sensitive information, and may invade stroke survivors’ homes [68]. However, it is crucial not to lose or diminish face-to-face contact due to increased use of ICT, especially during the initial stage of establishing a relationship with a stroke survivor [12]. In our study, digital technologies interfered with some therapeutic communication skills, especially during communication with individuals having depressive or cognitive impairments, such as aphasia. Nonetheless, being reflexive about the technological environment in which therapists work and its impact on clinical practice has been advocated [69], despite disagreements in rating the importance of factors related to therapists’ decision-making in implementing new technologies [61].

This study presented examples of therapists being unable to practice without certain technologies and concerns about losing therapeutic skills or positions owing to technology takeover. Concepts like technological determinism and disruption indicate that technologies shape societal structures and practices [70,71]. Similarly, the idea that new technologies disrupt social practices, norms, and even moral concepts has been revisited in the context of 21st-century technologies, particularly AI [71]. These concepts imply a reduced role for agents in changing practices, which we do not acknowledge, and this rather points back to the distribution of responsibility. In this study, we identified examples of technology that could shift responsibility and change routines. Therefore, new therapeutic communication skills are essential to adapt technology to people [70,72].

Assessments of stroke survivors’ functional levels are needed not just in the initial phase but throughout rehabilitation to evaluate necessary adjustments. However, consideration of assessment accuracy must be taken into account [53]. Research supports that assessment is an important feature from the perspective of HCPs when using digital technologies in stroke rehabilitation [57,73]. In this study, digital monitoring enabled closer follow-up on progress and increased stroke survivors’ self-management through collaboration with therapists. Shared understanding and priorities can promote technology implementation, including understanding its role relative to conventional therapy, training models, and evidence-based practice [52].

In motor rehabilitation, digital technologies appeared more complicated owing to the principles of intensity and repetition, which often require in-person assistance. While stroke rehabilitation may be too complex for full digitalization, partial implementation is feasible, especially combined with in-person support. Studies have found significant effectiveness of VR for varied outcomes [74], particularly for improving cognitive function compared with conventional care in a hospital setting [75]. Digital technologies for motor rehabilitation might be effective in a hospital setting but might face limitations in home environments [74]. In a previous study, participants continued the same types of exercises at home, transferring skills and preserving performance [75], which HCPs in our study noted as challenging. Additionally, studies have cautioned against the misfit between participants’ needs and available technology, emphasizing the importance of coherent content and use to avoid misunderstandings and misuse in practice [32,53]. This approach depends on the stroke trajectory and the practice setting, as individuals may experience different recovery paths and require tailored interventions based on the stroke onset and the environment in which they receive rehabilitation [53]. The diverse perceptions between the 2 practices in this study appear to be related to engaging with people at different stages of stroke onset in the cross-section of hospitalization and home rehabilitation. The consideration of a continuous digital transition that follows stroke trajectories and becomes more self-managed in the chronic phase seems appropriate.

Researching rehabilitation practices raises questions about what is involved in stroke rehabilitation. Motor rehabilitation, as described by Kwakkel et al [43], involves a process that enhances the motor function, activity capacity, and daily performance of stroke survivors, aligning with the International Classification of Functioning, Disability and Health (ICF) framework [76]. Rehabilitation extends beyond restoring function, aiming to maximize independence and facilitate participation in home and community life [77]. Our study highlights varied perceptions of rehabilitation, underlining its multidisciplinary nature and the importance of coordinated and cross-professional contribution [32]. A digitalized rehabilitation service will require changes in the workflows of professionals, fostering cross-disciplinary collaboration to build capacity and ensure greater availability across time and settings. This shift will challenge traditional professional boundaries while breaking down silos between researchers and clinicians [78].

### Strengths and Limitations

The data and findings in this study are unique as we applied social practice theory, which allowed us to comprehensively analyze the current and potential future influence of digital technologies for HCPs in poststroke rehabilitation and increased our awareness and reflexive interpretation of the contextual factors influencing these practices. It is acknowledged that the successful implementation of digital technologies depends on not only their inherent qualities but also how they are adopted and integrated into existing practices by HCPs. Maintaining an explorative approach, the findings are broad and consistent with the complexity and comprehensiveness of rehabilitation. A potential limitation of this study is the extended data collection period, which spanned 2 years but was divided into specific recruitment timepoints. While this allowed for a broader capture of experiences and perspectives, shifts in practice or external factors during this period may have influenced the findings. Some digital technologies, such as videoconference systems, VR tools, and webinar tools, were more frequently integrated into HCPs’ daily practices at the first site during an earlier recruitment period (2022‐2023), while step counters were more regularly used at the second site. These differences may reflect evolving practices influenced by project-based initiatives and specific time periods. These temporal variations should be considered when interpreting the results. Representatives of most multidisciplinary HCPs in specialized stroke rehabilitation participated in this study, with the majority recruited from a hospital rather than a community-based clinic. The inclusion of specialist stroke rehabilitation hospital wards provides a more specific context. As the participants emphasized cross-service collaboration and community collaboration, reflections from community therapists could have added nuances to the findings. No psychologists participated as individual professionals, and only 1 HCP with a background in psychology took part. Future research could investigate the perspectives of psychologists and mental health specialists to expand the theme of psychosocial support and better tailor digital technologies to the psychological and emotional needs of stroke survivors. This study focused on the statements of HCPs regarding how they performed practices, while an ethnographic study would have provided detailed information about the implicit social structures in a specialized stroke rehabilitation practice. The use of RTA allowed for deep engagement with the data, enhancing transparency in the research process. Moreover, the collaboration between experienced researchers and clinicians ensured a robust and informed analysis, strengthening the study’s credibility. Although this study covered current and proposed future technologies, it might have missed emerging innovations or future novel digital tools that the participants might not have been aware of, such as generative AI and robotics, which could potentially revolutionize practices. The study acknowledged challenges in stroke survivor follow-up continuity after referral, suggesting that future research is needed to explore detailed solutions and develop strategies for effective digital integration in rehabilitation.

### Conclusion

This study found that HCPs used different technologies during their daily specialist care practices, as well as for the coordination and follow-up of stroke survivors after referral to community services. While all practice themes influenced rehabilitation practices, ambivalence and challenges were noted, particularly in digitalizing multidisciplinary psychosocial support and exercise programs. Despite ambivalence, digital technologies can serve as valuable supplements for stroke recovery, enhancing accessibility, competence, information flow, and quality care. This study identified several factors, such as organizational processes, roles, standards, and rules, that can act as barriers or drivers to implementing digital technologies in practices, emphasizing coherence among content (thing), use (agent), and aim (rules). Systems for sharing medical records and goal-setting apps, which are aimed at enhancing coordination and stroke survivor involvement, were emphasized as future digital technologies that can shape stroke rehabilitation. Viewing technology as a supplement to existing practices, rather than a singular solution for all areas of specialized stroke rehabilitation, offers significant potential for quality improvement. By focusing on leveraging already familiar technologies, specialized stroke rehabilitation integrates agent skills, physical tools, and practice guidelines, forming strategies to enhance the use and effectiveness of digital technologies in the field. Our findings may inform technology developers, health care personnel, and user groups in specialized neurological rehabilitation settings.

## Supplementary material

10.2196/77753 Multimedia Appendix 1 Interview guide.

10.2196/77753 Multimedia Appendix 2 Mapping of qualitative data.

10.2196/77753 Multimedia Appendix 3 Six phases of reflexive thematic analysis.

## Abbreviations

AI: artificial intelligence
AR: augmented reality
GP: general practitioner
HCP: health care professional
ICT: information and communication technology
NAV: Norwegian Labor and Welfare Administration
RTA: reflexive thematic analysis
SMART: specific, measurable, achievable, relevant, and time-bound
VR: virtual reality
WHO: World Health Organization
