# Supplementary material for: Exploring the Influence of Digitalization on Multidisciplinary Poststroke Rehabilitation Practice: Qualitative Study
Source: JMIR Rehabil Assist Technol. 2026 Feb 17;13:e77753. doi: 10.2196/77753 (PMC12912659; doi:10.2196/77753)
Supplement: Multimedia Appendix 1 [file rehab-v13-e77753-s001.docx]

**Multimedia Appendix 1.** Interview guide.

**INTERVIEW GUIDE for healthcare professionals**

Participant's name and code: ________________________________________
Gender:________________________________________________________
Age:___________________________________________________________
Native language:_________________________________________________
Rehabilitation institution:___________________________________________

**Understanding the healthcare professional’s practices at the rehabilitation institution**

1. How is post-stroke rehabilitation practice conducted here? / Can you describe the rehabilitation of a stroke patient you remember well?
2. What is most important for you to achieve in the rehabilitation of stroke patients?
3. What clinical guidelines are used at your practice?
4. What measurement tools/clinical tests are used at your practice?

**Investigating user involvement in stroke rehabilitation**

1. How did you and the patient collaborate in the rehabilitation process?
2. What information did you provide for the patient?

**How are digital technologies (such as mobile apps, digital blood pressure monitors, heart rate, pedometers, or information and communication technology) used in stroke rehabilitation?**

1. What kinds of digital technologies (on mobile phones, PCs, iPads) are currently being used at your practice?
2. How have digital technologies influenced your practice?

**Investigating future development of digital technologies in rehabilitation**

1. Do you think / how do you think digital technologies can be useful in the rehabilitation process?
2. What are your wishes for digital advancements that could be used in stroke rehabilitation?
3. How do you think digital technologies can assist in rehabilitation?
4. Is there anything else you would add about your experiences with digital technologies in stroke rehabilitation?
