# Supplementary material for: Exploring the Influence of Digitalization on Multidisciplinary Poststroke Rehabilitation Practice: Qualitative Study
Source: JMIR Rehabil Assist Technol. 2026 Feb 17;13:e77753. doi: 10.2196/77753 (PMC12912659; doi:10.2196/77753)
Supplement: Multimedia Appendix 2 [file rehab-v13-e77753-s002.pdf]

PRACTICE	THINGS	AGENTS
Goal-setting conversations (Now)	**HCP do goal-setting conversations (especially in the anamnesis and pre-assessments) by clarifying realistic expectations and outcomes of the patient's condition to settle the strategies and plans for the rehabilitation process.**	**The conversation is conducted with empathy, respect, and sensitivity to help the patient's in formulating concrete and realistic goals to avoid frustrations in further life.**
Goal-setting conversations (Future)<br>Total references in codes	**Apps with push-warnings for goal-setting.**	**Communicate on goal-setting through digital interaction.**
Follow-up and continuity in rehabilitati	**HCP use videoconference (digital conversations) to evaluate further follow-up and need for hospitalisation with patients, informal caregivers, interpreters, or municipalities and less in further follow-up after hospitalisation unless it is other HCP.**	**In using digital services the need for HCP with digital competence is valued important.**

Follow-up and continuity in rehabilitation between the HCP and patient (Future)<br>Total reference in codes	**Possibilities of sending medical reports to cooperating instances, but also in direct communication with patients regarding their schedules and further goals.**	**In digital follow-up of patients after hospitalisation the therpeutic skills of being understanding yet with clear anticipations for the patient is addressed. A new type of agent is introduced - the therapist with NAV information.**
Assessments and monitoring of progres	**In assessments the HCP often use instrument to measure pulse during rehabilitation.**	**The importance of assessment for HCP is to do different assessment to act upon them.**
Assessments and monitoring of progres<br>Total reference in codes	**Outpatient consultations using videoconference to assess the patients' environment.**	**Using (local) ressources in the municipalities to aid pre-assessments before hospitalisation.**

Communication and psychosocial sup[illegible]	**Setting up structured support conversations or assessing quality of life or having unstructured conversations.**	**Importance of creating a relation and continuing the support by active listenting, being open, and sharing throughout the stroke process also in digital follow-up. Therapists' communication skills enable sensitive information from patients.**
Communication and psychosocial sup[illegible] Total reference in codes	**Supporting patients and informal caregivers with more digital follow-up in the later rehabilitation stage.**	**The importance of relationship building before further digital support conversations.**
Involve patients in their rehabilitation ([illegible]	**Involve patients in discussions in several meeting points.**	**Stroke patients must lead their rehabilitation, and how stroke-related impairments or non-native speaker might undermine accountability.**

Involve patients in their rehabilitation (t<br>Total reference in codes	**Patients will be more involved in discussions at meetings in the future if all the steps in the digital follow-up and rehabilitation involves patients.**	**Need for more active and seeking patients.**
Exercises and rehabilitation programs (	**Gamified rehabilitation.**	**Therapy-assisted training at distance or present.**
Exercises and rehabilitation programs (<br>Total reference in codes	**Rehabiltation programs either self-managed or supported with less complex patients (e.g. dysarthria or upper-limb impairments).**	**Being goal-oriented and progressive.**

Collaborations between services and c	**There is lack of knowlegde on the available "things" in the wider stroke practice.**	**It is resource-intensive for both the patient and subsequent healthcare personnel to manage, for example, goals and rehabilitation plans during the transition between rehabilitation institutions and home to the municipalities, particularly for patients recovering from conditions that have affected their cognitive functioning rather than mobility.**
Collaborations between services and c<br>Total reference in codes	**Educating in electronic individual plan.**	**Learners from other hospitals**.
Navigating patients in the care pathway	**HCP initiate the local opportunities to continue rehabilitation.**	**HCP emphasise pre-conversations (consultations) as they provide valuable information and better prepare the patients. The demands to patients might vary in between institutions or even wards. Patients are often referred to other rehabilitation institutions because they need more comprehensive rehabilitation.**

Navigating patients in the care pathway (future)	**More video-conference in navigating patients in care pathway.**	**Cognitive and motor adjustments.**
Total reference in codes		
Involving next of kin/informal caregivers (now)	**Conversations with caregivers in meetings or after reminder notes.**	**Support caregivers.**
Involving next of kin/informal caregivers (future)	**More written material.**	**Joint digital competence between patient and caregiver.**
Total reference in codes		

RULES/DISCOURSE	PROCESS/STRUCTURE
**Healthcare professionals routinely use SMART goals and the expectation clarifications in conversations with patients to establish rules.**	**Setting goals is giving directions for future rehab and continuosly evaluated by focusing on patient's own daily task-related goals framed together with therapists using the SMART principle.**
**The continued and routine way of understanding goal setting could be enabled by apps, but HCP also question that there migth be a differece between goalrelated intervention and the patients's daily goals.**	**Healthcare professionals imagine the extention of practice could be enabled by digital monitoring and analyzing health-related data and goals that specifically focuses on factors contributing to health and well-being (salutogen surveillance).** 103
**The norm is to use digital pre-assessments conversations and policlinic conversations and it is HCPs duty ensure a further follow-up.**	**In the follow-up most digital communication is used, and less virtual training. Still there is need to strenghten the digital communication for instance in communicating through individual plan.**

There is societical incitaments of increasing the outpatient activity and outsources information to the patient, which is valued among therapists.	Re-structuring the practice so there is better access to stroke-related information, a joint health-record system for communication, and logistics.	90
Detailed mapping, a holistic understanding of the patient's needs, and ongoing evaluation to assess effectiveness are underlying norms.	Assessment as an indicator of therapeutic needs and gaining a holistic view of rehabilitation.	
Feedback system for screening and motivation.	Monitoring for assessing performance.	66

To advice in life situations of loss (of self) and depression, which are harder doing digitally.	HCP often have conversations with patients in all phases post-stroke because (the consensus among 50 %) rehabilitation is about managing the new self. Also, patients with multiple cognitive and psychiatric challenges often requires more conversations and is experienced as difficult patients to engage with.
The responsibility of the therapist in support follow-up.	Relation-building through conversation acts as a prerequisite for digital support. 77
The aim and rule of involving patients in rehabilitation is to take care own existential needs. The goal is for patients to become as independent as possible, encouraging them to be active.	Adjusting based on individualised needs. Challenges of providing patient-centered care in a rehabilitation setting (particularly for patients with cognitive impairments), but with close collaboration between HCP.

**Balancing the norm of active patients and the lack of total insight and comprehension.**	**Preserving the therapeutic codex in procedures.The importance of patient-centred or individualised approaches in rehabilitation.** 121
**There are different "rules" around dosage of training, but the direction is turning to high-intensity training and whole-body exercises instead of isolated movements.**	**Use the training potential.**
**Facilitate support and environment.**	**Integrate technology into the rehabilitation process while maintaining a high standard of individualized adaptation and considerations on treatment effectiveness (flexible follow-up, support, simple technology).** 122

Accommodate the various expectations and norms from the specialist healthcare services to the municipal level, as well as the patient's understanding of rehabilitation at the system level.	Patients and healthcare providers face challenges in process (transition) from rehabilitation institutions to home or community care. HCP emphasize the need for better coordination between rehabilitation institutions and community care providers, more resources and training for community care.
A norm in co-creation between institutions and communication across other HCP.	HCP and patient need more information on rehabilitation options in the process. 77
Rehabilitation priorities is based on patient's needs and rehabilitation potential. Many patients return to the same rehabilitation institution for follow-up and meeting other needs, also because the availability of for instance occupational therapy is lacking in some municipalities.	A normal prosedure is discussing patients between HCP. Sometimes complex cases demand a stronger guide from therapists in helping patients achieve their goals, providing clear understandings of therapy plan and emphasise the role of therapists knowing what’s best for the patients.

Sometimes different rules undermine seamless digital transitions and the spread of reaching people digitally.	Division of responsibilities and resources in collaboration between different levels of healthcare personnel. 82
Balancing the need for support from caregivers and involving professional help.	Involving patients and caregivers help establish a foundation enabling them feeling safe.
The majority is digitally proficient.	Support network for future practice. 77
