# Supplementary material for: Exploring the Influence of Digitalization on Multidisciplinary Poststroke Rehabilitation Practice: Qualitative Study
Source: JMIR Rehabil Assist Technol. 2026 Feb 17;13:e77753. doi: 10.2196/77753 (PMC12912659; doi:10.2196/77753)
Supplement: Multimedia Appendix 3 [file rehab-v13-e77753-s003.docx]

| **Familiarising** | - Read transcripts several times and wrote reflexive, rough notes with initial interpretations - Identified variations in specialised post-stroke rehabilitation practices across the HCP dataset - Observed that data varied by professional roles, with rehabilitation spanning monitoring, close assessments, return-to-work, and intensive, task-varied physical rehabilitation - Noted practice conflicts both across and within sectors, highlighting the need for a theoretical framework to guide latent, deductive analysis - Refined the working research question, providing clearer direction for coding |
| --- | --- |
| **Coding** | - Transcript were read line-by-line, and initial codes were written using NVivo - Began inductive coding while reading Social Practice Theory (SPT) by Reckwitz, Lloyd, Hargreaves, and Schatzki - Conducted two rounds of coding: first, annotations were written on how the empirical material aligned with SPT - Refined codes and revisited transcripts to ensure no codes were prematurely conceptualised with SPT terms, avoiding theoretical bias or affirming evidence prematurely - Example codes related to the themes:   **Digital coordination**  Digital interaction, pre-conversations for user involvement, directed user involvement, information flow (internal and external), conflicts between institutions, municipality-to-specialist care flow, phase-based goal-setting, autonomy, knowledge sharing, and digital goal-setting  **Digital actions in conversations**  Digital vs. therapeutic conversations, dialogue-based technology, challenges with user involvement and aphasia, ethical conversations, digital therapeutic adaptation, quality-of-life mapping, time for individuals with greater needs, impersonal nature of digital communication, and relational significance in rehabilitation  **Digital training**  Robotic technology, digital assessment, virtual training, challenges digitising physiotherapy, therapist as functional promoter, rehabilitation as screening, goal-setting, and training, valued training quantity, changes in motor approaches, simulated training, specialists in assessment, training beyond high intensity, and re-testing for control |
| **Generating initial themes** | - Before generating initial themes, we wrote reflexive interpretations to describe each code. - Examined and grouped similar codes to identify broader patterns of meaning (potential themes). - Grouped codes in a table without assigning theme names initially, then began to develop preliminary themes. - Framed theme titles in the first person, imagining participants expressing them to ensure alignment with the dataset and participants' perspectives. - Initially, four themes were generated:   1. Digital tools for continuity and coordination of rehabilitation   2. Psychosocial support versus digital communication   3. Digital supplements in assessments and physical rehabilitation   4. Navigating user-centred approaches in the digital era |
| **Developing and reviewing themes** | - After coding and theme generation using RTA, a second analysis refined themes and linked them to the four Social Practice Theory (SPT) concepts, examining both current and future healthcare practices in Excel. - This analysis produced a directory of themes and sub-themes, with participant quotations organized under each SPT concept and sub-theme heading. - Theme development considered the entire dataset to refine theme boundaries and deepen interpretation. - Initial themes were checked against coded data and the full dataset to ensure they told a convincing and coherent story addressing the research question. - Quotations for each initial theme were combined with the four SPT concepts to guide theme refinement. - Themes were divided into sub-themes, aligning with the SPT concepts. - Codes were revisited and restructured where necessary to ensure they were grouped appropriately. |
| **Refining, defining and naming themes** | - The dual analysis approach captured both individual and collective dimensions of practices, ensuring conceptual and theoretical generalisability. - This phase involved refining each theme by defining its scope, focus, and overarching ‘story.’ - Informative and descriptive names were assigned to each theme. - In the SPT analysis, descriptions were written for each sub-theme linked to SPT concepts, which further refined and evolved the initial theme names |
| **Writing up** | - Writing the manuscript and refining results and discussion with co-authors finalised the analysis. - This phase involved integrating the analytic narrative with data extracts and situating the analysis within existing literature. - Several rounds of writing and discussion led to adjustments in theme boundaries, identification of latent patterns, and exploration of theme relationships. - Quotations were revisited in their original context to ensure they accurately reflected participants' intent |
